# Respiratory efficiency and thermoregulatory responses of L-citrulline-supplemented broiler chickens under acute and chronic stress conditions

**DOI:** 10.3389/fphys.2026.1785584

**Published:** 2026-04-21

**Authors:** Lan Li, Victoria Anthony Uyanga, Hongchao Jiao, Jingpeng Zhao, Xiaojuan Wang, Haifang Li, Koukou Tona, Hai Lin

**Affiliations:** 1 Key Laboratory of Efficient Utilization of Non-grain Feed Resources Co-construction by Ministry and Province, Ministry of Agriculture and Rural Affairs, Shandong Agricultural University, Taian, Shandong, China; 2 Shandong Provincial Key Laboratory of Animal Nutrition and Efficient Feeding, College of Animal Science and Technology, Shandong Agricultural University, Taian, Shandong, China; 3 Department of Agriculture and Environmental Sciences, Lincoln University of Missouri, Jefferson, MO, United States; 4 College of Life Sciences, Shandong Agricultural University, Taian, Shandong, China; 5 Regional Center of Excellence for Avian Sciences, University of Lome, Lome, Togo

**Keywords:** broilers, heat stress, immune stress, L-citrulline, lipopolysaccharide, thermoregulation

## Abstract

**Introduction:**

Poultry birds are exposed to diverse environmental stressors, including high ambient temperatures and endotoxins, which negatively affect the birds’ health and productivity. This study investigated the impacts of both stressors and the mediatory role of dietary L-citrulline (LCT) on the physiological responses of broiler chickens.

**Methods:**

A total of 384, 1-day-old broiler chicks were randomly divided into 4 groups, 8 replicates of 12 chicks, and fed two dietary treatments of basal diet (CON) or basal diet supplemented with 1% LCT. At 21 days old, broilers were subjected to acute conditions of heat stress (HT:35 ℃ vs thermoneutral (TNZ:24 ℃); experiment 1) or immune stress (2 mg/kg BW lipopolysaccharide (LPS) or saline; experiment 2) and monitored for 5hours in a respiratory chamber. In the chronic LPS challenge (experiment 3), birds at 41days old were administered 1 mg/kg BW LPS at 1, 6, and 24 hours.

**Results and discussion:**

Exposure to HT conditions elevated the body temperature, O2 consumption, CO2 expiration, and heat production (HP) of broilers (P>0.05). Broilers fed the LCT diet also exhibited increased O2 consumption, CO2 expiration, and HP relative to the CON diet (P>0.05). Contrastingly, acute LPS challenge reduced the O2 consumption, CO2 expiration and HP. The provision of dietary LCT tended to reduce the core body temperature (CBT) of HT broilers and exerted significant effects by decreasing the CBT of LPS-challenged broilers (P>0.05). LCT supplementation also diminished the respiratory quotient (RQ) of broilers exposed to either HT or LPS compared to the unchallenged LCT groups, which had elevated RQ (P>0.05). The plasma inducible nitric oxide synthase levels were reduced by the LCT diet, with a consequent decline in plasma NO concentrations in experiment 3. Examining the mRNA expression of thermosensing TRP ion channels revealed that HT upregulated hypothalamic TRPV1 expression, whereas chronic LPS downregulated skin TRPV2 and TRPA1 expressions. In addition, the co-treatment of LPS with LCT further evoked a decline in skin TRPA1 expressions (P>0.05).

**Conclusion:**

Overall, this work demonstrated the capacity of HT and LPS challenge to dysregulate whole-body metabolism and the potential of dietary LCT to exert mediatory effects, which may be crucial for the reestablishment of homeostasis.

## 1 Introduction

Heat stress caused by high environmental temperatures is a serious problem in poultry production, especially in the tropical and subtropical regions where birds frequently encounter elevated summer temperatures (Xu et al., 2023). Heat stress raises the body temperature of poultry and triggers a cascade of physiological responses (Chowdhury et al., 2014). It occurs when the heat generated by an animal exceeds its ability to dissipate to its surrounding environment. Under high-temperature conditions, broiler chickens are particularly susceptible to heat stress because they lack sweat glands, are covered with feathers, and have a rapid growth rate and high metabolism (Chowdhury, 2023; Uyanga et al., 2022a). Heat stress induces a range of physiological alterations, including oxidative stress, disruptions in acid-base equilibrium, gut dysbiosis, and reduced immune function (Wasti et al., 2020). These changes can subsequently result in higher mortality rates, decreased feed efficiency, lower body weight, reduced feed intake, diminished egg production, and compromised meat and egg quality (Azad et al., 2010).

L-citrulline is a non-essential, α-amino acid that serves as an endogenous precursor to L-arginine. L-citrulline is known to play an important role in blood pressure, vasodilation, anti-inflammation, protein synthesis, intestinal homeostasis, anti-oxidation, renal function, exercise performance, and pharmaconutrition (Morita et al., 2014; Uyanga et al., 2022a). Recent findings show that L-citrulline can reduce body temperature, relieve heat stress, and improve thermotolerance of chickens (Chowdhury et al., 2017; Uyanga et al., 2021, 2022b). The arginine-nitric oxide cycle allows for the endogenous synthesis of nitric oxide (NO) from arginine (Arg), with L-citrulline as a byproduct. L-citrulline can be recycled back to L-arginine via the sequential actions of arginosuccinate synthase and arginosuccinate lyase enzymes (Uyanga et al., 2023). NO synthesis from arginine is catalyzed by three isoforms of nitric oxide synthase (NOS): endothelial NOS (eNOS), neuronal NOS (nNOS), and inducible NOS (iNOS) (Förstermann and Sessa, 2012). NO generated subsequently diffuses into the vascular smooth muscle cells where it acts a biological signaling molecule eliciting various roles, including vasodilation, neurotransmission and immune defense.

Additionally, NO is involved in controlling the thermogenic response during the inflammatory process, such as the fever response induced by lipopolysaccharide (Garami et al., 2018). Lipopolysaccharide (LPS), a crucial element in the cell walls of gram-negative bacteria is potent for mimicking inflammatory reactions and stress scenarios in poultry and livestock (Zhou et al., 2023). Both heat stress and LPS can activate immune responses and also influence the body’s response to thermoregulation (Goel, 2021). Research has found that animals treated with LPS exhibit more pronounced inflammatory responses when exposed to heat stress, specifically manifested as a significant increase in body temperature, and higher levels of interleukins and tumor necrosis factor (Lin et al., 2009).

Exposure to heat, endogenous ligands, mechanical, and osmotic stress may activate the transient receptor potential (TRP) ion channels, which act as sensory mediators in the regulation of normal and pathological functions in the vasculature during response to local environmental changes (Baylie and Brayden, 2011; Lamas et al., 2019). Once activated, these channels may contribute to vasodilation via NO, prostacyclin, and potassium channel-dependent pathways (Baylie and Brayden, 2011). They can be found as heat sensitive ion channels with varying activation thresholds in different tissues, such as the transient receptor potential vanilloid 1 (TRPV1; > 43°C) (Lamas et al., 2019); and TRPV2 (> 52°C), which is predominantly expressed within specific brain regions such as the cerebellum, forebrain, and hippocampus (Lamas et al., 2019). The TRPV3 is a temperature-sensitive transient receptor potential ion channel, with an activation temperature range of 32~40°C (Desai and Clapham, 2005), while the TRPV4 functions as a non-selective cation channel capable of detecting temperature variations within the range of 27~ 34°C (Liedtke et al., 2000). In contrast, the TRPM8 was discovered as an ion channel capable of activation at temperatures below 25°C (Lamas et al., 2019). Similarly, the TRPA1 can be activated at cooler temperatures, responding to environmental conditions as low as 17°C (Story et al., 2003). The activation of the TRPA1 channel through depolarization is regarded as a critical mechanism that triggers the skin vasodilation responses during exposure to cold temperatures (Aubdool et al., 2014). To date, researchers have identified these six thermosensitive TRP ion channels: TRPV1, TRPV2, TRPV3, TRPV4, TRPA1, and TRPM8, with each of these channels exhibits a unique range for temperature sensing (Dhaka et al., 2006).

This study investigated the effects of L-citrulline on the body temperature regulation, whole-body metabolism, and immune response of broiler chickens managed using stress models of high ambient temperatures and LPS-induced immune dysfunction. The study aimed to elucidate the role of L-citrulline in thermoregulation through examining the changes to the basal metabolism, activation of NO-generation, and regulation of TRP ion channels.

## 2 Materials and methods

This research was performed in accordance with the “Guidelines for Experimental Animals” of the Ministry of Science and Technology (Beijing, P. R. China), and the study protocols were approved by the Institutional Animal Care and Use Committee of the Shandong Agricultural University, China.

### 2.1 Experimental animals

1-day-old male broiler chicks (Arbor Acres) were purchased from a commercial hatchery (Xintai Liuhe Breeding Co., Ltd., Taian, Shandong) and raised in three-tiered battery cages with a cage dimension of 1.40 m × 0.70 m × 0.38 m. The environmental temperature was 35°C on the first day, and decreased by 1°C every other day, to 24°C at 22 day, and then remained unchanged. The humidity was kept at 60 ± 5% throughout the rearing period (**Supplementary Figure 1**). The light regime was 23h light (1:00~24:00):1h dark (0:00~1:00). The birds had free access to feed and water during the study, except as mentioned otherwise, and the nutrient composition of the basal diet is shown in **Table 1**.

**Table 1:** Nutrient composition of basal diets fed to broiler chickens.

Ingredient	Starter diet (%, 0-21d)	Grower diet (%, 22-42d)
Corn (8.3% CP)	56.98	62.23
Soybean meal (43% CP)	30.46	24.75
Corn gluten meal (57.5% CP)	5.00	5.00
Corn starch	1.00	1.00
Soybean oil	2.37	3.04
Limestone	1.40	1.37
CaHPO_4_	1.58	1.47
Salt	0.32	0.30
Lysine (99%)	0.23	0.26
Methionine (98%)	0.15	0.12
Choline chloride (50%)	0.26	0.20
Vitamin premix ^1^	0.05	0.05
Trace element premix ^2^	0.20	0.20
Total	100	100
Nutrient composition		
Crude protein %	21.0	19.0
Metabolizable energy, kcal/kg	3000	3100
Calcium %	0.95	0.90
Available phosphorus %	0.44	0.41
Salt %	0.35	0.32
Lysine %	1.100	1.000
Methionine %	0.500	0.450
Methionine + Cysteine %	0.857	0.780
Arginine %	1.268	1.110

^1^The vitamin premix provides the following quantities per kilogram of diet: vitamin A, 8000 IU; vitamin D_3_, 1000 IU; vitamin K, 0.5 mg; vitamin E, 20 IU; VB_1_, 2mg; riboflavin, 8 mg; D-pantothenic acid, 10 mg; VB_5_, 35 mg; VB_6_, 3.5 mg; VB_12_, 0.01mg; biotin, 0.18 mg; folic acid, 0.55mg; choline, 1300mg.

^2^Mineral premix provides the following per kg of diet: Fe (as ferrous sulfate), 100 mg; Zn (as zinc sulfate), 100 mg; Mn (as manganese sulfate), 120 mg; Cu (as copper sulfate) 8 mg, I (as potassium iodide), 0.7 mg; and Se (as sodium selenite), 0.3mg.

### 2.2 Experimental design

A total of 384 day-old broiler chickens with similar body weight (36.87 ± 0.18g) were randomly assigned into two treatments of 16 groups, and 12 chickens per group. The chicks were fed with either a basal diet (CON group) or the basal diet supplemented with 1% L-citrulline (LCT group), which was purchased from Shandong Fosun Biotechnology Co., Ltd., China (**Table 1**). The L-citrulline dosage was determined based on previous experiments that showed that this dose reduced core body temperature in broilers under heat stress, promoted NO synthesis, and increased plasma immunoglobulin levels (Uyanga et al., 2022c, 2022d). Broilers were implanted with a core body thermometer (DS1922L, Maxim, CA, USA) at 14 days of age, then used for subsequent experiments.

### 2.3 Heat stress model

At 18 days old, 36 chickens were selected from each treatment (n=72) and transferred to 12 respiratory chambers containing 6 birds each, with individual cage dimensions of 900×1400×1100 mm (Shandong Mingjun Ecology Technology Co., Ltd., China). During the 3-day habituation period in the respiratory chamber, broilers were allowed free access to feed and water for acclimatization.

For the acute heat stress study (experiment 1) at 21 days of age, birds were arranged using a 2 by 2 factorial design having two dietary treatment (CON vs LCT) and two environmental temperature (TNZ- 24°C vs HT-35°C). Prior to the trial, feed was withdrawn, and the environmental temperature was set to 24°C (CON + TNZ group, LCT + TNZ group) and 35°C (CON+HT group, LCT+HT group).

**Figure 1:**
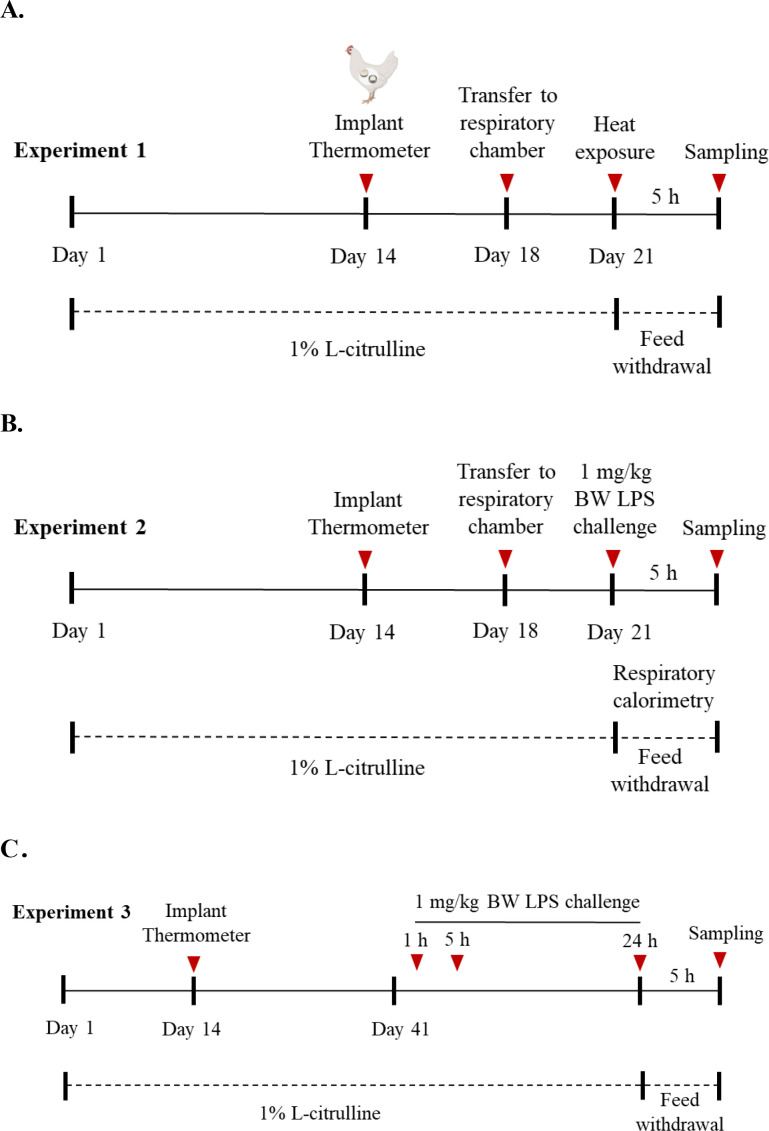
Schematic layout of experimental designs. **(A)** Acute heat stress (experiment 1); **(B)** Acute lipopolysaccharide challenge terminated at 21-days of age (experiment 2); **(C)** Chronic lipopolysaccharide challenge terminated at 42 days of age (experiment 3).

The birds were monitored for 5 hours and O_2_ and CO_2_ concentrations were continuously measured. Thereafter, one chicken from each chamber was selected for blood and tissue collection. The flow chart of the experimental design is shown in **Figure 1A**.

### 2.4 Immune stress model

For the acute immune stress study (experiment 2), 24 chickens per treatment (n=48) were selected and randomly assigned using a 2 by 2 factorial arrangement of dietary treatment (CON vs LCT) and injections (Saline vs LPS), which resulted in four treatment groups of CON + Saline, CON+LPS, LCT + Saline, and LCT+LPS. In each treatment, two chambers were used, and 6 birds per chamber. The Saline group was injected intraperitoneally with saline (H37022336, CISEN Pharmaceutical Co., Ltd.) as a sham control, while the LPS group was injected with 2 mg/kg BW LPS (L6529, lipopolysaccharide from Escherichia coli, sigma-Aldrich) and then monitored at 24°C for 5 hours post-injection and O_2_ and CO_2_ concentrations were continuously measured. The flowchart of the experiment is shown in **Figure 1B**.

For the chronic immune challenge (experiment 3), 16 bird per treatment (n=32) was selected at 41 days of age and intraperitoneally injected with LPS (1 mg/kg BW) at 1, 6, and 24 hours to induce different stages of immune fever and inflammation. These chickens were arranged using a 2×2 factor design with two levels of dietary treatment (CON vs LCT) and two levels of injections (Saline vs LPS), similar to experiment 2. Thereafter, one chicken per replicate of treatment was selected for blood and tissue collection. The flowchart of the experiment 3 design is shown in **Figure 1C**. For the stress models, broilers at 21 and 41 days old were examined. At 21 days of age, the broiler’s immune system is initially mature, metabolism is vigorous but reserves are limited. This period is suitable for observing rapid and intense stress responses. The 42-day-old stage is the critical point for commercial broiler slaughter. At this point, the body has undergone long-term growth pressure and metabolic load, making it suitable for simulating the real stress conditions encountered in actual production. It is suitable for verifying the comprehensive effect of citrulline in alleviating chronic heat stress and protecting growth performance.

### 2.5 Growth performance

The body weight and feed intake of broilers in each replicate were recorded at 1, 21, and 42 days of age, and computed to determine the average daily gain (ADG), average daily feed intake (ADFI), and feed conversion ratio (FCR). These parameters were corrected for mortality.

### 2.6 Blood and tissue collection

At the end of each study, one chicken was randomly selected from each replicate for sampling (n=32). Blood was drawn from the wing vein of each chicken and collected into sodium heparin anticoagulant tubes. Plasma samples were obtained by centrifugation at 3500×*g* for 15 min at 4°C and stored at -20°C for further analysis. Broiler chickens were euthanized by cervical dislocation, which was verified by palpation, and exsanguination was done by severing the carotid artery (AVMA, 2020). Tissue samples including the hypothalamus and skin were isolated, rapidly frozen in liquid nitrogen, then stored at -80°C for further biochemical analysis.

### 2.7 Metabolic rate measurements

The respiratory heat production of broiler chickens in experiments 1 and 2 was measured in a respiratory chamber (Shandong Mingjun Ecological Agriculture Technology Co., Rizhao, Shandong, China) using the “open-circuit negative pressure”, as previously reported (Liu et al., 2024). Except under the acute stress model, the respiratory chamber was maintained at constant temperature and humidity of 24 (± 1)°C; 60% RH. The gases of oxygen (O_2_) and carbon dioxide (CO_2_) were continuously extracted from the respiratory chamber through a vacuum pump a rate of 20 L/min, and measured via the gas analyzer at 3-minute intervals. The fasting heat production and respiratory quotient were computed as.

Heat production (Kcal) = 16.1753×VO_2_+5.0208×VCO_2_
Heat production/BW (Kcal) = HP/BW^0.75^
Where VO_2_ and VCO_2_ indicate the volumes of oxygen and carbon dioxide, respectively.

### 2.8 Core body temperature measurement

The core body temperature was recorded using an iButton digital temperature logger (DS1922L, Maxim, CA, USA). The recording time was preset, and the recording was made at 30-minute intervals. At 14 day old, the iButton was implanted into the abdominal cavity of broilers through laparotomy for the continuous monitoring of core body temperature. Data were obtained using the TMEX Viewer software (Shanghai Vodisen Electronic Technology Co., Ltd., China).

### 2.9 Surface temperature

The surface temperature was measured using an infrared thermal imager (TP 160 Suzhou Shengguang Instrument Co. Ltd., China). At a horizontal distance of about 1 m from the head and at the same height as the broiler chicken, the infrared thermal imager was used to capture the point temperature data of the ear of the broiler chicken. The accuracy of the thermal imager is ± 0.1°C, the wavelength is 7.5 ~ 13 μm, and the data obtained were analyzed using the FLIR Thermocam Researcher Pro 2.10™ (FLIR Systems Company, China) software.

### 2.10 Rectal temperature

Rectal temperature was measured using a KRUUSE Vet digital thermometer (Cat No. 291110, China). The probe of the rectal thermometer was inserted into the broiler’s cloaca to a depth of 1 to 3 cm, and the thermometer had a resolution of 0.1°C.

### 2.11 Nitric oxide concentration

The plasma nitric oxide (NO) concentration was measured using a commercial kit (A012-1; Jiancheng Bioengineering Institute, Nanjing, Jiangsu, China). NO is chemically reactive and is rapidly metabolized to NO2¯ and NO3¯ in the body, and NO2¯ is further converted to NO3¯. This method uses nitrate reductase to specifically reduce NO3¯ to NO2¯, and the concentration of NO2¯ was determined. The reaction absorbance was read at 550nm using a microplate reader (Elx808, Bio-Tek, Winooski, VT).

### 2.12 Nitric oxide synthase activity

The total NOS (tNOS) and inducible NOS (iNOS) were determined using a commercial assay kit (A01-1; Jiancheng Institute of Biological Engineering, Nanjing, Jiangsu, China) according to the manufacturer’s instructions. The optical density for tNOS and iNOS were measured with a 1cm cuvette at 530nm using a UV-2450 spectrophotometer (Beijing PGeneral, Beijing, China). The endothelial NOS (eNOS) activity was measured using a commercial ELISA kit (m1295147; Shanghai Enzyme-Linked Biotechnology Co., Ltd., Shanghai, China), and the absorbance was determined by a microplate reader (Elx808, Bio-Tek, Winooski, VT).

### 2.13 RNA extraction and quantitative real-time PCR analysis

The total RNA was extracted from ~100 mg of tissue using trizol reagent (NCM Biotech, China) according to the manufacturer’s guidelines. The RNA concentration was measured using a nucleic acid spectrophotometer (Eppendorf, Germany), with A260/A280 ratio ≥ 1.8 -2.01 indicating high RNA purity. Reverse transcription was done using the HiFiScript cDNA Synthesis Kit according to manufacturer’s guidelines. Real-time polymerase chain reaction was performed using the Fast SYBR Green Master Mix in 20μl mix per reaction on the ABI7500 fluorescence quantitative polymerase chain reaction system (Applied Biosystems, Bedford, USA). The reaction conditions were: 95°C reaction for 10 min, 95°C reaction for 10 s, 60°C reaction for 20 s, 72°C reaction for 30 s, for a total of 40 cycles. The relative expression of the target gene mRNA was analyzed by 2^-ΔΔCT^method, and the primer sequences used for RT q-PCR are shown in **Table 2**.

**Table 2:** Primers used for RT-qPCR.

Gene	GeneBank No.	Orientation	Primer sequence (5´→3´)	Product length, bp
TRPV1	XM_046929895.1	Forward Reverse	GATGGATCACCTGATGGCACCTTC TCAGAGCAGCCTGTGATGGAGTC	105
TRPV2	XM_040687364.2	Forward Reverse	AGCAACCAAAAGGACCCCAATCG CAAGTAAGCCATCCAGTGCCTCAG	91
TRPV3	XM_040687357.2	Forward Reverse	TCAATGCAGCATACACCGAAGAGG AAAGAAAATACCCTGGGCGTGAGC	140
TRPV4	NM_204692.2	Forward Reverse	AGCAAGATTGAGAACCGCCATGAG AGGAGACCACGCTGATGTAGAAGG	112
TRPM8	XM_046921262.1	Forward Reverse	GCACCGCTGGGAGTGGATATTTC ACGCACAAGGGTTTGGACTCATTC	144
TRPA1	NM_001318460.2	Forward Reverse	CCGTTCCTGAGTTACACCGTTCTG TCCCCAACAGCCAAACCAATTAGC	89
GADPH	NM_204305.2	Forward Reverse	CTACACACGGACACTTCAAG ACAAACATGGGGGCATCAG	244

TRPV 1-4, Transient receptor potential vanilloid 1-4; TRPM8, Transient receptor potential cation channel subfamily M (melastatin) member 8, TRPA1, Transient receptor potential cation channel, subfamily A, member 1.

### 2.14 Statistical analysis

Data were analyzed using statistical analysis software (SAS, version 8e, SAS Institute, Cary, NC). Data collected were analyzed as a 2 × 2 factorial arrangement of diet (CON vs LCT) and temperature (TNZ vs HT) for the acute heat stress model or diet (CON vs LCT) and injection (Saline vs LPS) for the immune stress models to obtain the main effects of diet, treatments and interactions. Where interaction effect was non-significant, the main effects of diet or treatments were also analyzed using T-test. Rectal and core body temperature data were analyzed using a three-factor repeated measures ANOVA to assess the main effects of diet, injection, and time, as well as their interactions. *P* < 0.05 was considered to be significant, and *P* < 0.1 was regarded as a tendency towards significance. Significant differences between means were assessed by Duncan’s multiple range test. All results are expressed as means ± standard error, and charts were generated using GraphPad Prism (GraphPad Software, La Jolla, CA, United States).

## 3 Results

### 3.1 Growth performance of broilers

**Table 3** shows that compared to the CON diet, supplementation with dietary LCT did not significantly affect the ADFI, ADG and FCR of broilers at 1 to 21 d of age (*P>*0.05). However, at the grower phase of 22 to 42d, the ADFI and ADG were significantly decreased in the LCT-fed broilers compared to the CON group (*P* < 0.05). The overall growth performance from 1 to 42 d revealed that the LCT-fed broilers had decreased ADG and 42d BW compared to the CON diet (*P* < 0.05).

**Table 3:** Effect of dietary L-citrulline supplementation on the production performance of broilers.

	CON	LCT	*P-*value
BW(g)			
1d	36.90 ± 0.17	36.87 ± 0.18	0.918
21d	745.51 ± 14.16	760.22 ± 13.57	0.459
42d	2186.39 ± 36.20^a^	1994.64 ± 37.20^b^	0.0009
1-21d			
ADFI, g/d	40.40 ± 0.92	42.61 ± 0.68	0.063
ADG, g/d	30.61 ± 0.61	32.04 ± 0.94	0.211
FCR	1.32 ± 0.02	1.34 ± 0.02	0.603
22-42d			
ADFI, g/d	141.13 ± 2.53^a^	132.51 ± 2.68^b^	0.026
ADG, g/d	71.25 ± 1.92^a^	62.65 ± 1.70^b^	0.002
FCR	2.11 ± 0.07	2.17 ± 0.06	0.518
1-42d			
ADFI, g/d	91.14 ± 2.17	87.74 ± 1.62	0.120
ADG, g/d	50.94 ± 1.01^a^	47.31 ± 0.93^b^	0.013
FCR	1.72 ± 0.04	1.75 ± 0.04	0.566

Data were presented as the mean ± SEM (n =8). Means with different superscripts in the same row differ significantly (*P* < 0.05). CON, Basal diet; LCT, 1% L-citrulline added diet; ADFI, Average daily feed intake; BW, Bodyweight; ADG, Average daily gain; FCR, Feed conversion ratio.

### 3.2 Body temperature and plasma metabolites of broiler chickens subjected to acute heat stress (experiment 1)

**Figure 2:**
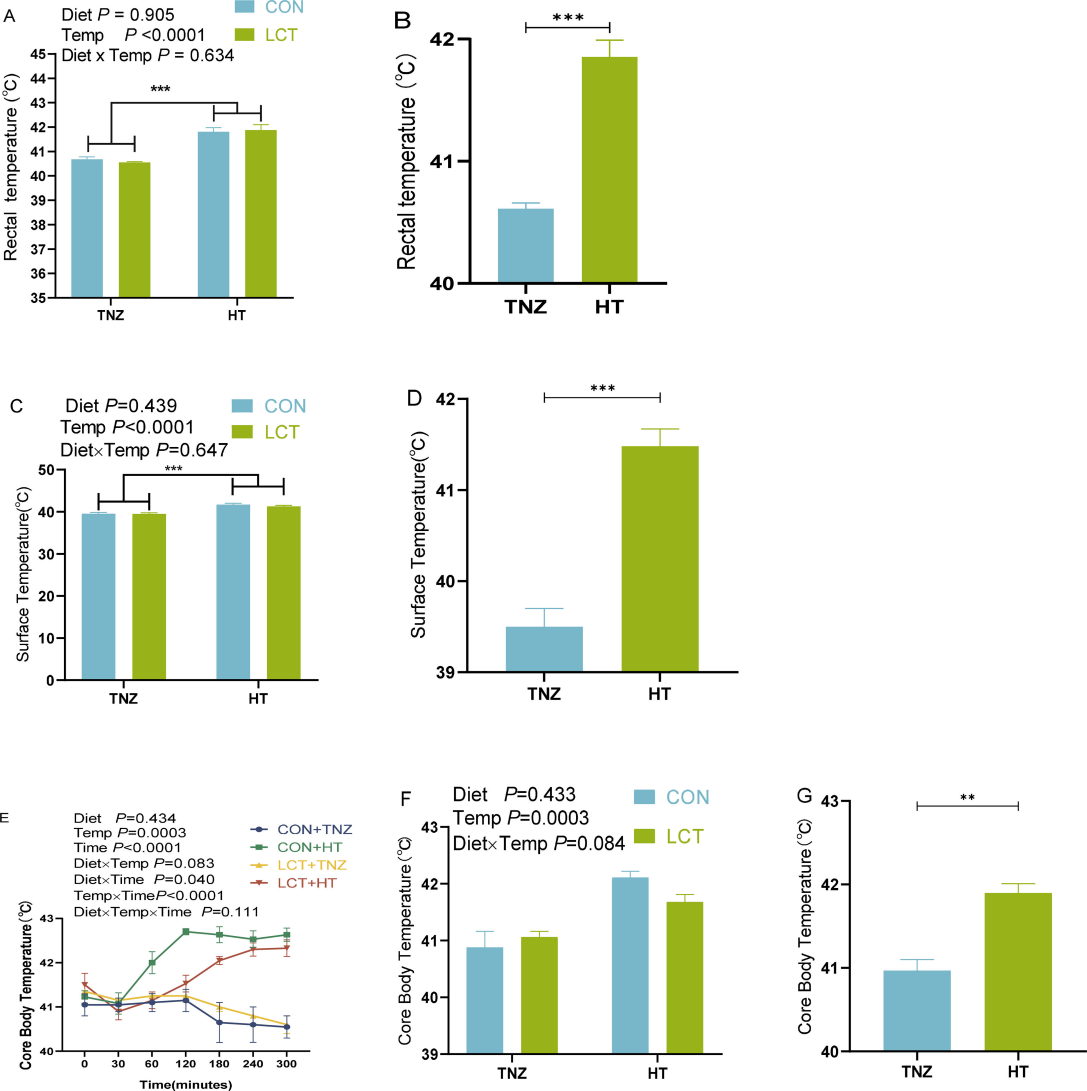
Body temperature changes of broiler chickens fed with dietary L-Citrulline and subjected to acute heat stress in experiment 1. **(A, B)** Rectal temperature, **(C, D)** Surface temperature, **(E–G)** Core body temperature. Data were presented as the mean ± SEM (n=8). ***indicates significant difference of treatment at *P* < 0.0001, and **indicates significant difference of treatment at *P* < 0.001. TNZ, Control temperature of 24°C; HT, High temperature of 35°C; CON, Basal diet; LCT, 1% L-citrulline added diet; Diet, Main effect of diet; Temp, main effect of temperature; Diet x Temp, Interaction effect of diet and temperature.

Under the condition of acute heat stress, the rectal, surface, and core body temperature of broiler chickens were significantly higher in the HT group relative to the TNZ group (**Figures 2A–G**). The core body temperature began to increase 30 minutes after HT exposure and remained elevated throughout the study (**Figure 2E**). Importantly, there was a significant impact of Time (*P* < 0.0001) as the core body temperature was elevated with prolonged duration in HT groups, and there was a tendency towards significance for Diet x Time (*P* = 0.0040). Numerically, this was evident in the elevated core body temperature for the CON + HT fed birds compared to the LCT + HT broilers, with a resulting difference of 0.18, 0.85, 1.17, 0.58, 0.23 ^0^C at 30, 60, 120, 180, 240, and 300 mins, respectively (**Figure 2E**). Overall, the LCT + HT group had ~0.43°C lesser core body temperature than the CON + HT birds, with a tendency towards statistical significance (**Figure 2F**, *P* = 0.084).

**Table 4** shows that there was no significant effect of diet by temperature interactions (*P*>0.05) on the NO concentrations and three NOS isoforms under acute heat stress. However, there was a significant main effect of Diet (*P =* 0.017*)* on iNOS activity, with the CON diet exhibiting higher iNOS activity compared to the LCT diet. The tNOS activity tended to decrease with the main effect of Temp (*P* = 0.075) since the HT groups had lower activity than the TNZ housed broilers. Interestingly, the plasma eNOS activity also showed an increasing activity under HT conditions, without any significant main or interaction effects (*P*>0.05).

**Table 4:** Effects of L-citrulline supplementation on plasma metabolites of broiler chickens subjected to acute heat stress (experiment 1).

	TNZ		HT		*P-value*		
	CON	LCT	CON	LCT	Diet	Temp	DietxTemp
NO (µmol/L)	60.05 ± 22.1	31.19 ± 4.66	47.81 ± 19.57	42.14 ± 6.60	0.316	0.970	0.498
tNOS (U/mL)	24.83 ± 5.08	19.09 ± 1.92	16.92 ± 1.26	18.87 ± 0.98	0.385	0.075	0.091
iNOS (U/mL)	4.94 ± 1.84	1.33 ± 0.31	1.95 ± 0.61	1.36 ± 0.38	0.017	0.083	0.075
eNOS (pg/mL)	125.71 ± 8.16	124.18 ± 9.98	142.50 ± 8.10	138.65 ± 8.62	0.783	0.119	0.905

Data were presented as the mean ± SEM (n =8). NO, Nitric oxide; tNOS, Total Nitric oxide synthase; iNOS, Inducible Nitric oxide synthase; eNOS, Endothelial Nitric oxide synthase, TNZ, Control temperature of 24°C; HT, High temperature of 35°C; CON, Basal diet; LCT, 1% L-citrulline added diet; Diet, Main effect of diet; Temp, main effect of temperature; Diet x Temp, Interaction effect of diet and temperature

### 3.3 Oxygen consumption and carbon dioxide expiration of broiler chickens subjected to acute heat stress (experiment 1)

**Figure 3:**
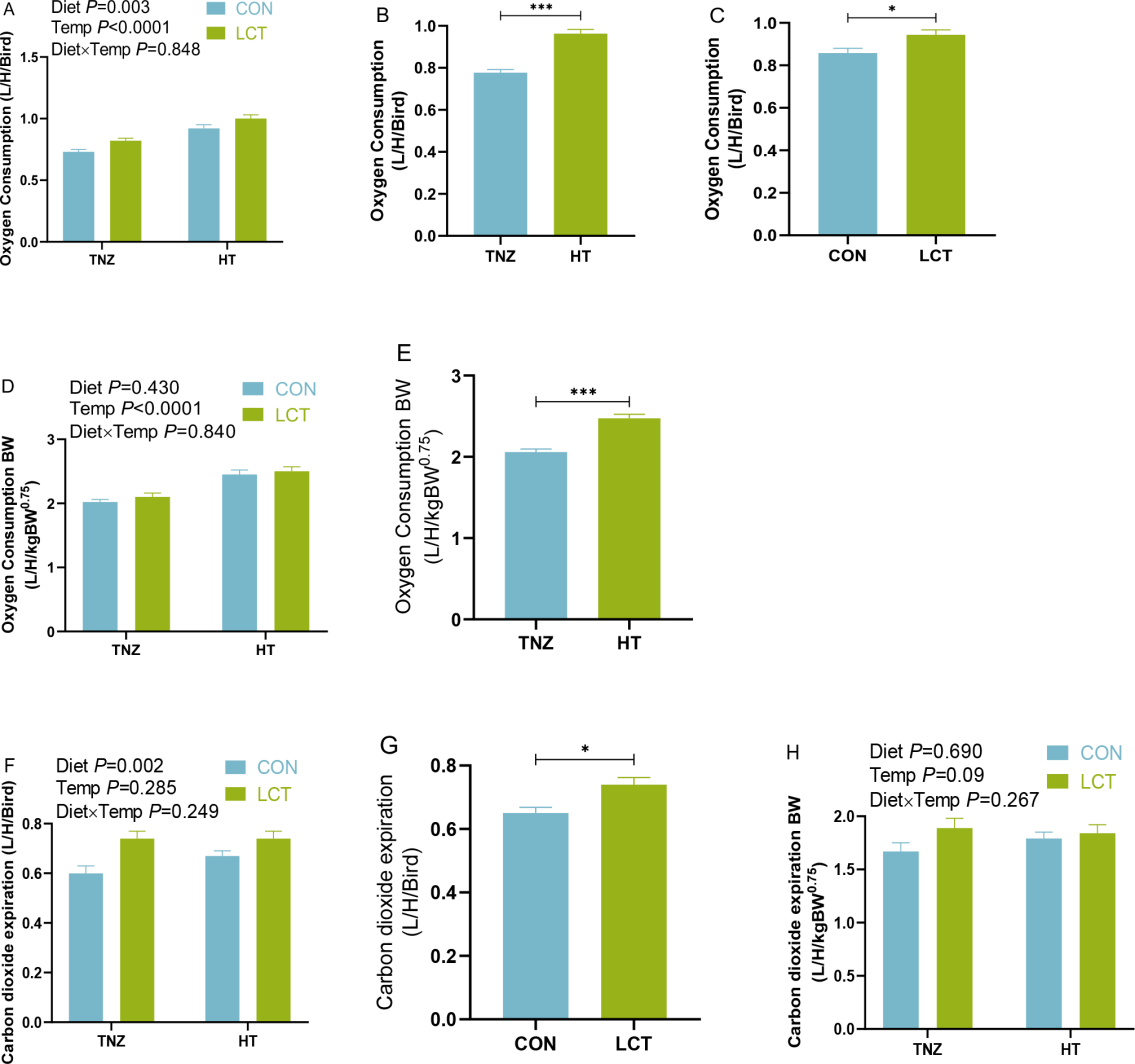
Oxygen consumption and carbon dioxide expiration rate of broilers under acute heat stress conditions of experiment 1 **(A–C)**. Oxygen consumption; **(D, E)**. Oxygen consumption expressed per metabolic BW; **(F, G)** Carbon dioxide expiration; **(H)** Carbon dioxide expiration expressed per metabolic BW. Data were presented as the mean ± SEM (n=8). Significant difference is indicated with asterisk as ***P < 0.0001, *P<0.05. TNZ, Control temperature of 24°C; HT, High temperature of 35°C; CON, Basal diet; LCT, 1% L-citrulline added diet; Diet, Main effect of diet; Temp, main effect of temperature; Diet x Temp, Interaction effect of diet and temperature.

Under acute stress conditions of experiment 1, there were no significant interaction effects (*P* > 0.05) of diet and temperature on the oxygen consumption and carbon dioxide expiration of broilers (**Figure 3**). The main effect of temperature was evident as broilers housed under HT conditions had increased oxygen consumption (**Figure 3B**), oxygen consumption per metabolic BW (**Figure 3E**), and carbon dioxide expiration (**Figure 3G**), compared to the TNZ housed broilers. Similarly, there was a significant main effect of diet (*P* = 0.003) as the LCT-fed hens had a higher oxygen consumption relative to their control-fed counterparts (**Figure 3C**).

### 3.4 Metabolic heat production and respiratory quotient of broiler chickens subjected to acute heat stress (experiment 1)

**Figure 4:**
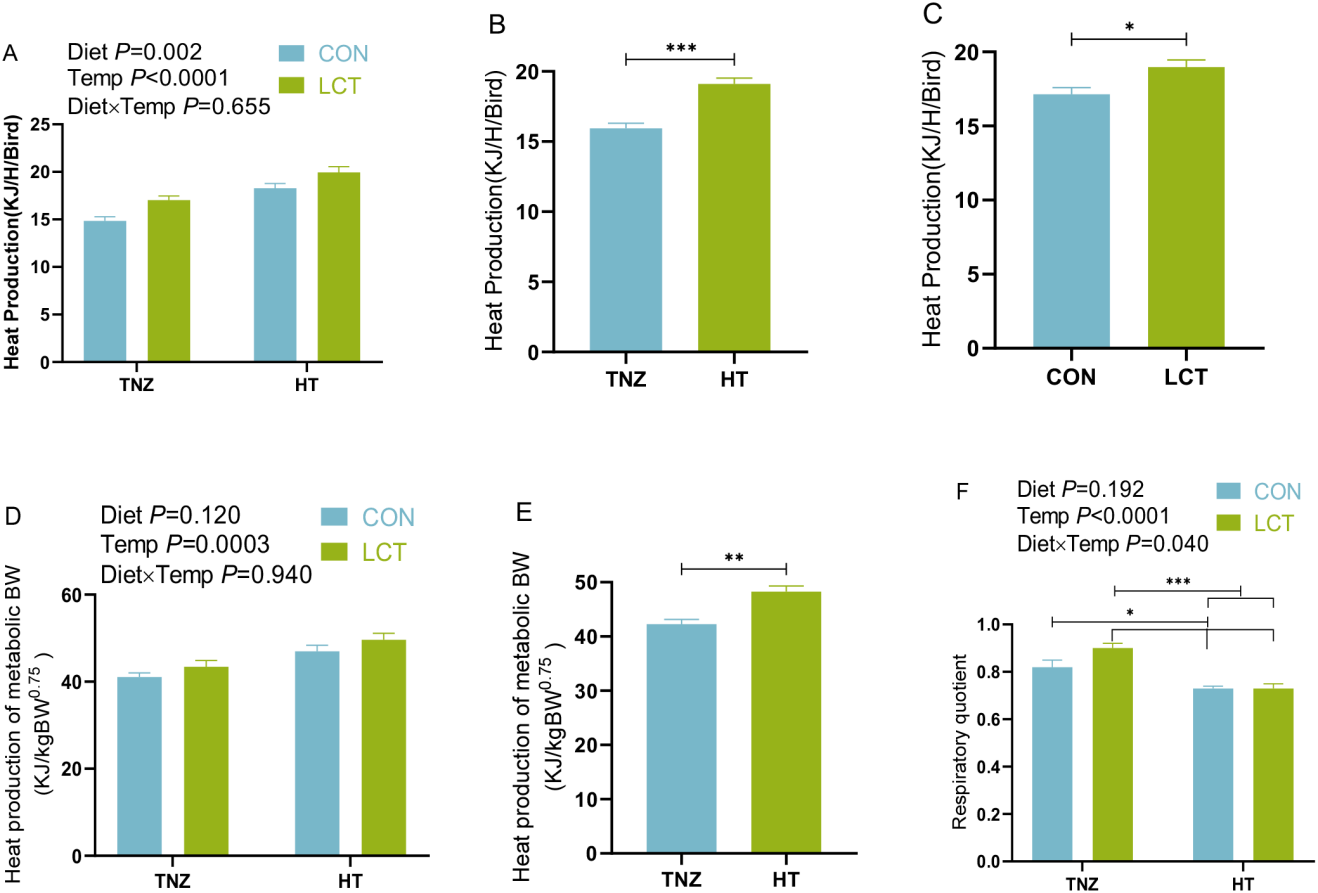
Metabolic heat production and respiratory quotient of broiler chickens subjected to acute heat stress conditions of experiment 1 **(A–C)**. Heat production **(D, E).** Heat production expressed per metabolic BW; **(F)** Respiratory quotient. Data were presented as the mean ± SEM (n=8). Significant difference is indicated with an asterisk as ***P < 0.0001, **P < 0.001, *P<0.05. TNZ, Control temperature of 24°C; HT, High temperature of 35°C; CON, Basal diet; LCT, 1% L-citrulline added diet; Diet, Main effect of diet; Temp, main effect of temperature; Diet x Temp, Interaction effect of diet and temperature.

Results show that the heat production and heat production expressed per metabolic BW were unaffected (*P* > 0.05) by interaction effects in experiment 1 (**Figure 4**). Notwithstanding, there was a significant main effect of temperature (*P* < 0.05), as broilers housed under the HT group had increased heat production (**Figure 4B**) and heat production per metabolic BW (**Figure 4E**) compared to the TNZ groups. Similarly, supplementing broilers with LCT diet significantly increased their heat production (*P* < 0.05), relative to the control fed birds (**Figure 4C**). Interestingly, the interaction of diet and temperature significantly (*P* < 0.05) influenced the respiratory quotient of broiler chickens, such that birds fed LCT under TNZ temperature had elevated respiratory quotient which was higher than other groups (*P* < 0.05). More so, the control-fed birds under TNZ housing had higher respiratory quotients compared to the HT housed birds that were fed either control or LCT diets (**Figure 4F**).

### 3.5 Relative mRNA expression of TRP ion channels in broiler chickens subjected to acute heat stress (experiment 1)

**Table 5** shows that there were no significant effects of diet by temperature interactions on the mRNA expression of TRP ion channels in the skin of broiler chickens. However, the main effect of diet was evident, such that the skin mRNA expression of TRPV1 tended to increase with LCT diet (*P =* 0.056), while TRPV3 expression was significantly upregulated by LCT diet (*P <* 0.05) compared to the CON diet. The skin mRNA expressions of TRPV1-4, TRPA-1, and TRPM8 were not significantly altered by the main effects of temperature (*P>*0.05).

**Table 5:** Effects of L-citrulline supplementation on the relative mRNA expression of TRP ion channels in the skin of broiler chickens subjected to acute heat stress (experiment 1).

	TNZ		HT		*P-value*		
	CON	LCT	CON	LCT	Diet	Temp	DietxTemp
Skin							
TRPV1	1.00 ± 0.26	2.57 ± 0.85	1.02 ± 0.22	1.46 ± 0.49	0.056	0.283	0.266
TRPV2	1.00 ± 0.55	1.8 ± 0.65	0.82 ± 0.18	1.21 ± 0.21	0.109	0.295	0.572
TRPV3	1.00 ± 0.26	2.55 ± 0.50	1.11 ± 0.21	1.59 ± 0.38	0.009	0.240	0.146
TRPV4	1.00 ± 0.37	1.63 ± 0.58	0.88 ± 0.26	1.16 ± 0.65	0.393	0.576	0.743
TRPA-1	1.00 ± 0.48	0.76 ± 0.05	0.55 ± 0.13	0.90 ± 0.29	0.843	0.583	0.288
TRPM8	1.00 ± 0.33	2.41 ± 0.79	1.79 ± 0.54	1.48 ± 0.55	0.369	0.906	0.172

Data were presented as the mean ± SEM (n=8). TRPV 1-4, Transient receptor potential vanilloid 1-4; TRPM8, Transient receptor potential cation channel subfamily M (melastatin) member 8, TRPA1, Transient receptor potential cation channel, subfamily A, member 1. TNZ, Control temperature of 24°C; HT, High temperature of 35°C; CON, Basal diet; LCT, 1% L-citrulline added diet; Diet, Main effect of diet; Temp, main effect of temperature; Diet x Temp, Interaction effect of diet and temperature.

**Table 6** shows that compared to the TNZ conditions, the hypothalamic TRPV1 mRNA expression was upregulated (*P <* 0.05) while TRPM8 also showed a tendency for an increase (*P =* 0.066) during HT exposure. In addition, there was a tendency towards significance due to the interaction effect (*P* = 0.066) on TRPM8 expression, with LCT diet decreasing TRPM8 expression under HT and TNZ conditions. Similarly, the LCT diet also tended to reduce TRPV3 expression compared to the CON diet (*P* = 0.073) in the hypothalamus of broilers. The hypothalamic expressions for TRPV2, TRPV4, and TRPA-1 were unaffected by diet, temperature, or interaction effects (*P>*0.05).

**Table 6:** Effects of L-citrulline supplementation on the relative mRNA expression of TRP ion channels in the hypothalamus of broiler chickens subjected to acute heat stress (experiment 1).

	TNZ		HT		*P-value*		
	CON	LCT	CON	LCT	Diet	Temp	DietxTemp
TRPV1	1.00 ± 0.24	0.35 ± 0.04	0.95 ± 0.08	0.76 ± 0.16	0.258	0.013	0.147
TRPV2	1.00 ± 0.08	0.97 ± 0.18	0.88 ± 0.06	1.13 ± 0.17	0.468	0.895	0.362
TRPV3	1.00 ± 0.17	0.54 ± 0.06	1.11 ± 0.08	0.91 ± 0.21	0.073	0.184	0.477
TRPV4	1.00 ± 0.35	0.33 ± 0.07	1.09 ± 0.27	1.27 ± 0.33	0.454	0.120	0.195
TRPA1	1.00 ± 0.15	0 62 ± 0.12	1.00 ± 0.15	1.01 ± 0.18	0.301	0.288	0.287
TRPM8	1.00 ± 0.28	0.26 ± 0.03	1.00 ± .014	0.98 ± 0.17	0.054	0.066	0.065

Data were presented as the mean ± SEM (n=8). TRPV 1-4, Transient receptor potential vanilloid 1-4; TRPM8, Transient receptor potential cation channel subfamily M (melastatin) member 8, TRPA1, Transient receptor potential cation channel, subfamily A, member 1. TNZ, Control temperature of 24°C; HT, High temperature of 35°C; CON, Basal diet; LCT, 1% L-citrulline added diet; Diet, Main effect of diet; Temp, main effect of temperature; Diet x Temp, Interaction effect of diet and temperature.

### 3.6 Body temperature of broiler chickens subjected to acute (experiment 2) and chronic (experiment 3) lipopolysaccharide challenge

**Figure 5:**
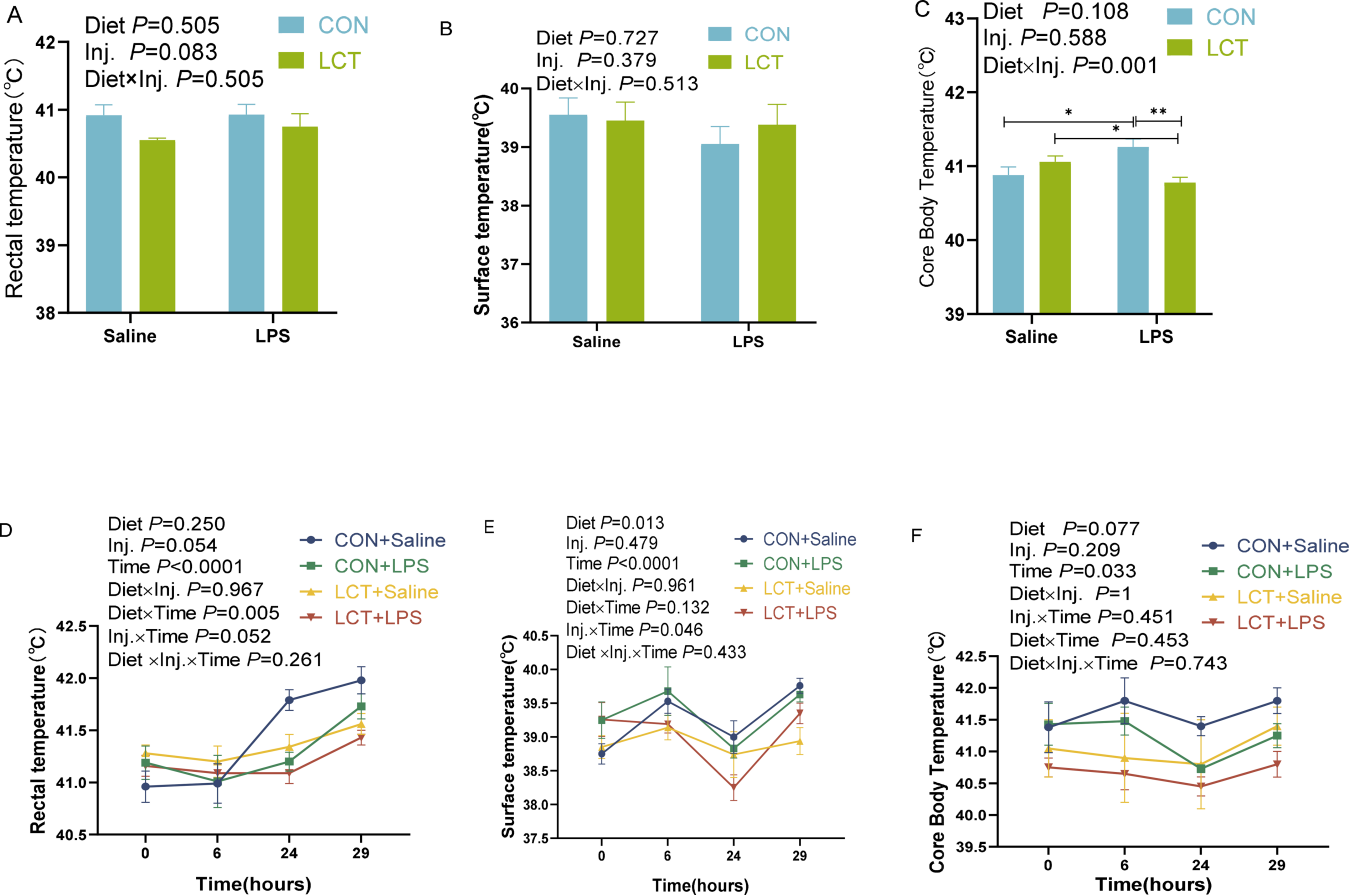
Effects of dietary supplementation of L-citrulline on body temperature of broilers subjected to lipopolysaccharide challenge of acute (experiment 2 at 21d) or chronic (experiment 3 at 42d) conditions. **(A)** Rectal temperature at 21 d **(B)** Surface temperature at 21d **(C)** Core body temperature at 21d **(D)** Rectal temperature at 42 d **(E)** Surface temperature at 42d **(F)** Core body temperature at 42d. Data were presented as the mean ± SEM (n=8). Significant difference is indicated on charts with asterisk as **P < 0.001, *P<0.05. LPS, Lipopolysaccharide; CON, Basal diet; LCT, 1% L-citrulline added diet; Diet, Main effect of diet; Inj., main effect of injection; Diet x Inj, Interaction effect of diet and injection.

Under acute LPS challenge, there were no significant interaction effects (*P* > 0.05) on the rectal and surface temperatures of broiler chickens (**Figures 5A, B**). However, the main effect of injection showed a tendency towards significance (*P* = 0.083) with LPS treatment inducing higher rectal temperatures. There was a significant interaction effect of diet and injection on the core body temperature of broilers (**Figure 5C**; *P* = 0.001) such that the LCT+LPS group had significantly lower core body temperature than the CON + LPS, and LCT + Saline groups. In addition, the CON+LPS broilers had higher core body temperature than the CON + Saline group (*P* < 0.05).

Under chronic LPS challenge, there was no significant three-way interaction effect of diet, injection, and time on the rectal, surface, and core body temperature of broiler chickens (**Figures 5D–F**). However, there was a significant two-way interaction effect of Diet × Time (*P* = 0.005) and Injection × Time (*P* = 0.052) on the rectal temperature of broilers (**Figure 5D**). The change in rectal temperature shows a significant time effect (*P* < 0.0001), which sustained the rise in rectal temperatures from 6h time point, until 29h post-injection (**Figure 5D**).

The surface temperature was significantly influenced by diet, time, and the interaction effect of injection and time (*P* < 0.05; **Figure 5E**). The changes in surface temperature revealed that the LCT-fed broilers had lower surface temperatures, which were consistent from 6 to 29h post-injection. The core body temperature of broilers declined steadily in the LCT +LPS relative to other groups, although there was no significant interaction effect (*P>*0.05; **Figure 5F**). In contrast, the CON +Saline group had the highest increments in core body temperature with a difference of 1.15, 0.95, and 1.00°C compared to the LCT +LPS at 6, 24 and 29h post-injection (**Figure 5F**). In addition, there was a significant main effect of time (*P* = 0.033) and a tendency for significance of dietary treatments (*P* = 0.077).

### 3.7 Oxygen consumption and carbon dioxide expiration of broiler chickens subjected to acute lipopolysaccharide challenge (experiment 2)

**Figure 6:**
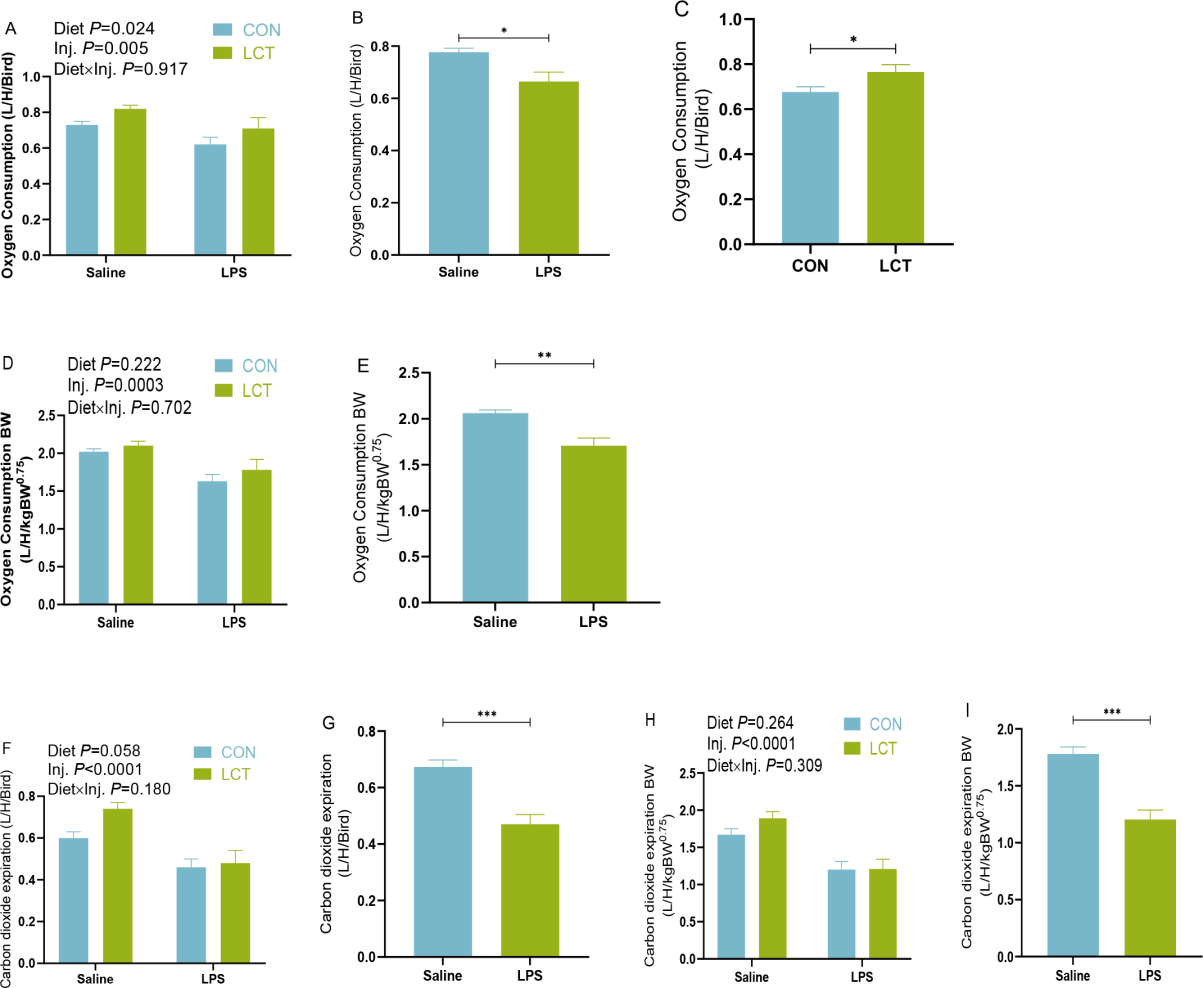
Oxygen consumption and carbon dioxide expiration rate of broilers subjected to acute lipopolysaccharide challenge in experiment 2. **(A–C)**. Oxygen consumption **(D, E).** Oxygen consumption expressed per metabolic BW; **(F, G)** Carbon dioxide expiration; **(H, I)** Carbon dioxide expiration expressed per metabolic BW. Data were presented as the mean ± SEM (n=8). Significant difference is indicated with an asterisk as ***P < 0.0001, **P < 0.001, *P<0.05. TNZ, Control temperature of 24°C; HT, High temperature of 35°C; CON, Basal diet; LCT, 1% L-citrulline added diet; Diet, Main effect of diet; Temp, main effect of temperature; Diet x Temp, Interaction effect of diet and temperature.

During acute immune stress, the oxygen consumption and carbon dioxide expiration of broilers chickens were unaffected by interaction effects (**Figure 6**). However, there was a significant main effect of injection (*P<*0.05), such that relative to the Saline group, LPS administration affected the respiratory efficiency of broilers by decreasing the oxygen consumption (**Figure 6B**), oxygen consumption per metabolic BW (**Figure 6E**), carbon dioxide expiration (**Figure 6E**), and carbon dioxide expiration per metabolic BW (**Figure 6I**). In addition, the main effect of diet (*P<*0.05), indicated that LCT fed broilers had higher oxygen consumption compared to the CON fed broilers (**Figure 6C**). Similarly, the carbon dioxide expiration was significantly influenced by the main effect of diet (*P =*0.058), such that LCT fed broilers had greater carbon dioxide expiration (**Figure 6F**).

### 3.8 Heat production and respiratory quotient of broiler chickens subjected to acute lipopolysaccharide challenge (experiment 2)

**Figure 7:**
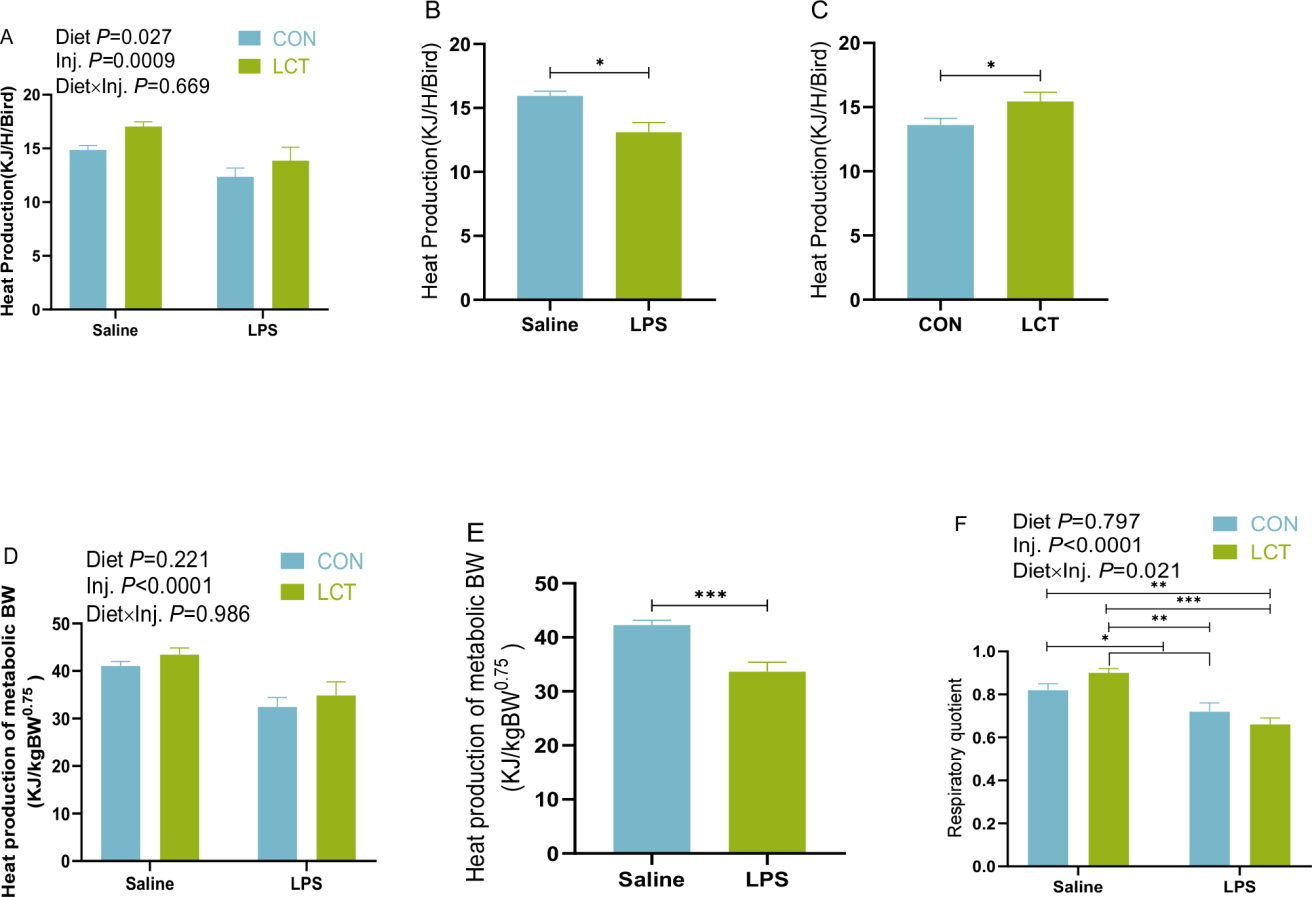
Metabolic heat production and respiratory quotient of broiler chickens subjected to acute lipopolysaccharide challenge of experiment 2. **(A–C)**. Heat production **(D, E)**. Heat production expressed per metabolic BW; **(F)** Respiratory quotient. Data were presented as the mean ± SEM (n=8). Significant difference is indicated with an asterisk as ***P < 0.0001, **P < 0.001, *P<0.05. TNZ, Control temperature of 24°C; HT, High temperature of 35°C; CON, Basal diet; LCT, 1% L-citrulline added diet; Diet, Main effect of diet; Temp, main effect of temperature; Diet x Temp, Interaction effect of diet and temperature.

There were no significant interaction effects of diet by injection on the heat production of broiler chickens (**Figure 7**). However, there were significant main effects of diet and injection, which influenced the metabolic heat production of broiler chickens. It was evident that LPS-treated broilers had lowered heat production (*P* < 0.05; **Figure 6B**), and heat production per metabolic BW (*P* < 0.05; **Figure 6E**) compared to the saline-injected broilers. More so, the main effect of diet significantly influenced the heat production (*P* < 0.05; **Figure 6A**), such that LCT fed chickens had lesser heat production than those fed CON diet (**Figure 6C**). Additionally, the respiratory quotient of broilers showed a significant interaction between diet and injection (*P* = 0.021), since LPS treated birds fed either CON or LCT diet exhibited decreased respiratory quotient compared to the Saline treated birds fed either CON or LCT diet (**Figure 6F**).

### 3.9 Plasma metabolites of broiler chickens subjected to chronic lipopolysaccharide challenge (experiment 3)

**Table 7** shows that during the chronic LPS challenge, the interaction of diet x injection had no significant effect on plasma NO concentrations and the activities of NO synthases (*P*>0.05). However, there was a significant main effect of dietary treatment on NO levels (*P* = 0.0003) and iNOS activity (*P* = 0.010). Treatment with dietary LCT decreased both the NO and iNOS levels compared to the CON diet. More so, LPS-injected birds had decreased iNOS activity relative to the saline group (*P <*0.05).

**Table 7:** Effects of L-citrulline supplementation on plasma metabolites of broiler chickens subjected to chronic Lipopolysaccharide challenge (experiment 3) conditions.

	Saline		LPS		*P-value*		
	CON	LCT	CON	LCT	Diet	Inj.	Diet x Inj.
NO (µmol/L)	57.5 ± 5.29	40.00 ± 5.47	73.25 ± 5.94	44.00 ± 5.84	0.0003	0.091	0.307
tNOS (U/mL)	27.47 ± 1.20	26.20 ± 0.48	27.67 ± 1.20	26.56 ± 0.44	0.170	0.738	0.922
iNOS (U/mL)	15.51 ± 1.46	11.66 ± 1.94	8.49 ± 1.48	4.39 ± 0.32	0.010	<0.0001	0.929
eNOS (pg/mL)	137.34 ± 6.00	130.98 ± 2.38	138.35 ± 4.99	132.82 ± 2.20	0.171	0.738	0.922

Data were presented as the mean ± SEM (n=8). Means with different superscripts in the same row differ significantly (P < 0.05). NO, Nitric oxide; tNOS, Total Nitric oxide synthase; iNOS, Inducible Nitric oxide synthase; eNOS, Endothelial Nitric oxide synthase. LPS, Lipopolysaccharide; CON, Basal diet; LCT, 1% L-citrulline added diet; Diet, Main effect of diet; Inj., main effect of injection; Diet x Inj, Interaction effect of diet and injection.

### 3.10 Relative mRNA expression of TRP ion channels in broiler chickens subjected to chronic lipopolysaccharide challenge (experiment 3)

Under chronic immune challenge, there was a significant interaction effect of diet and injection that influenced the skin mRNA expression for TRPV3 (*P* = 0.028) and TRPA1 (*P* = 0.012), with TRPV1 having a tendency (*P* = 0.082) towards significance (**Table 8**). The mRNA expression of TRPV3 in the CON + Saline group was significantly higher than that in CON + LPS (*P* < 0.05). Similarly, the expression level of TRPA1 in the CON + Saline group was significantly higher than in the other treatment groups (*P* < 0.05). The main effect of injection influenced TRPV2 expression such that compared with Saline, the LPS injection significantly downregulated the expression of TRPV2 (*P* = 0.024) in the skin. The expressions of TRPV4 and TRPM8 in the hypothalamus were not affected by diet, injection, or interaction (*P*>0.05). 

**Table 8:** Effects of L-citrulline supplementation on the relative mRNA expression of TRP ion channels in the skin of broiler chickens subjected to chronic lipopolysaccharide challenge (experiment 3) conditions.

	Saline		LPS		*P*-value		
	CON	LCT	CON	LCT	Diet	Inj.	Diet x Inj.
TRPV1	1.00 ± 0.36	0.67 ± 0.21	0.88 ± 0.23	1.77 ± 0.48	0.410	0.161	0.082
TRPV2	1.00 ± 0.29	0.88 ± 0.22	0.25 ± 0.07	0.65 ± 0.16	0.490	0.024	0.215
TRPV3	1.00 ± 0.33^a^	0.47 ± 0.12^ab^	0.26 ± 0.05^b^	0.61 ± 0.15^ab^	0.631	0.134	0.028
TRPV4	1.00 ± 0.44	0.58 ± 0.24	0.37 ± 0.11	0.63 ± 0.25	0.783	0.326	0.245
TRPA1	1.00 ± 0.20^a^	0.33 ± 0.09^b^	0.29 ± 0.11^b^	0.34 ± 0.10^b^	0.027	0.013	0.012
TRPM8	1.00 ± 0.37	0.58 ± 0.15	0.51 ± 0.13	0.72 ± 0.21	0.658	0.471	0.194

Data were presented as the mean ± SEM (n=8). Means with different superscripts in the same row differ significantly (*P* < 0.05). TRPV 1-4, Transient receptor potential vanilloid 1-4; TRPM8, Transient receptor potential cation channel subfamily M (melastatin) member 8, TRPA1, Transient receptor potential cation channel, subfamily A, member 1. LPS, Lipopolysaccharide; CON, Basal diet; LCT, 1% L-citrulline added diet; Diet, Main effect of diet; Inj., main effect of injection; Diet x Inj, Interaction effect of diet and injection

## 4 Discussion

The present study investigated the effects of dietary L-citrulline supplementation on the thermoregulation and metabolic rate of broiler chickens under stress conditions. In this work, heat stress and lipopolysaccharide-induced immune challenge were employed as stress models, as they have both been proven to influence body temperature regulation, basal metabolic rates, and inflammatory responses in poultry (Candido et al., 2020; Gray et al., 2005; Uyanga et al., 2022b). Heat stress exposure is typically accompanied by a series of physiological, metabolic, and behavioral adaptive responses triggered by the imbalance between heat production and heat dissipation in animals (Roushdy et al., 2020). In the present study, broiler chickens housed under acute heat stress conditions exhibited elevated rectal, skin, and core body temperatures, indicating a disturbed thermal balance. These findings corroborate previous reports that during heat stress, the body cannot effectively dissipate excess heat, causing its internal temperature to rise (He et al., 2019; Kim et al., 2025).

Consequently, the basal metabolic rate of the birds was assessed during their exposure to environmental heat stress by examining the oxygen consumption, carbon dioxide expiration, heat production, and respiratory quotient (RQ). These indices were further expressed per unit of metabolic body weight to account for the non-linear relationship between body size and metabolic rate. This standardization allows for fair comparisons across different body sizes, ages, or species, and improves the accuracy of physiological and nutritional assessments by normalizing metabolic differences based on Kleiber’s law (Martinez et al., 2022; Morillo et al., 2023). The industry standard for broiler weight at market release is approximately 2.6-2.8 kg, with a feed-to-meat ratio of 1.6-1.8. Our results show that the FCR was 1.72 - 1.75, which complies with the commercial standards (Aviagen, 2014). The final body weight at d42 was lower than the commercial standard, which may be attributed to compounding factors that the birds may have been exposed to during the trial. Importantly, adequate periods of recovery and acclimatization were provided throughout the study; however, these deviations in the performance of experimental birds are noted as a limitation of the current study. In this study, L-citrulline-fed broilers had lower final body weights and average daily gains, which contradicts our earlier report that L-citrulline improved the body weight gain of broilers and alleviated heat stress-induced suppression of body weight and feed intake at certain intervals (Uyanga et al., 2022c). Recent studies have also reported on the differential growth responses to L-citrulline, which may result in an indifference (Ashtary-Larky et al., 2025), improvements (Ma et al., 2025) or reduction (Kudo et al., 2021) in body weight and body weight gain of animals. These variations may be related to its role in energy and lipid metabolism, gut microbial modulation, and route of administration. Importantly, it highlights the complexity and biological variability of L-citrulline metabolism under health and stress conditions.

Broilers under acute heat stress exhibited elevated oxygen consumption, heat production, heat production per metabolic BW, and respiratory quotients. It was evident that broilers had higher oxygen consumption during heat stress because they relied on panting (increased breathing rate) for evaporative cooling, which elevated their oxygen demand. Birds activate heat-dissipating mechanisms (e.g., vasodilation, wing spreading) to thermoregulate; these processes require more ATP, thus increasing cellular respiration and oxygen use. These changes are aimed at survival and thermoregulation, but prolonged oxygen demand under heat stress can lead to metabolic imbalance, oxidative damage, and reduced performance (Gonzalez-Rivas et al., 2020).

Heat stress causes significant alterations in tissue substrate metabolism, which typically results in an increased energy expenditure and decreased RQ (Ma et al., 2021). In the present study, broilers managed under thermoneutral temperatures had higher RQ values, with the L-citrulline-fed broilers having an RQ of ~0.90, which was slightly higher than the control diet of ~0.82, suggestive of carbohydrate metabolism. Contrarily, the RQ value of heat-stressed broilers was lowered to about ~0.73 regardless of L-citrulline supplementation, suggesting a shift towards lipid metabolism. The RQ is the ratio of metabolic carbon dioxide production to oxygen intake, which is an index that helps determine the key metabolic substrate oxidized during cellular respiration, with a ratio between 0.7 and 0.8 indicating the utilization of lipids and proteins as energy substrates, whereas an RQ greater than 0.8 indicates the use of carbohydrates as the main energy source (Serano et al., 2022).

The findings of this work present an interesting relationship between the body temperature and the metabolic rate of chickens, with broilers exhibiting higher RQ under thermoneutrality, whereas reduced RQ and greater reliance on lipid catabolism occur during heat stress. In addition, it is plausible that heat stress-induced reductions in feed intake and reduced blood flow to the gastrointestinal tract may impair nutrient absorption and decrease metabolic energy input, thereby lowering the respiratory quotient of heat-stressed birds (McKee et al., 1997). In the present study, heat-stressed broilers had greater oxygen consumption and heat production, but lowered RQ. The elevated metabolic activity and shifts in metabolism may increase the oxygen requirement for energy production. This directly aligns with previous studies where an increased oxygen consumption with reduced RQ in heat-stressed mice was regarded as a marker of hypermetabolism (Serano et al., 2022).

Lipopolysaccharides (LPS) are a key component of the bacterial cell wall, which is closely associated with the activation of inflammatory signaling pathways and the onset of immune distress and inflammatory responses (Yang et al., 2024). In poultry, LPS administration is known to induce a rise in body temperature and initiate a febrile response, which is mediated by the synthesis of prostaglandins and nitric oxide (Gray et al., 2005). As such, LPS administration directly contributes as an exogenous pyrogen with immunosuppressive effects, making it a suitable model for the study of avian thermoregulation and body metabolism. In the present study, acute LPS challenge in experiment 2 elevated the core body temperatures of broilers greater than those of the Saline-treated group, although this febrile response was suppressed in the L-citrulline-fed broilers. Studies have shown that chicks exposed to LPS attacks often exhibit a regulatory hypothermia rather than fever as their initial response. This process is accompanied by a significant inhibition of the metabolic rate, namely a decrease in oxygen consumption (do Amaral-Silva et al., 2022). In this study, after acute injection of lipopolysaccharide, the respiratory quotient of chicks changes significantly due to immune activation. It was found that after the injection of lipopolysaccharide, the carbon dioxide emissions, oxygen consumption, respiratory entropy, body heat production, and metabolic heat production per unit weight of the chicks all significantly decreased, which is consistent with the existing research findings. During chronic LPS challenge (experiment 3), the rectal, surface, and core body temperatures of broilers were influenced by significant time-dependent changes; however, there were no significant impacts of LPS challenge. The differential response between acute and chronic LPS response may be a typical endotoxin tolerance pattern in which there is a reduced responsiveness to LPS challenge following an initial encounter with the endotoxin. LPS tolerance is regarded as a natural reprogramming of the immune system that increases the number of immune cells and decreases the production of cytokines (Melo et al., 2009).

An assessment of the basal metabolism during acute LPS-challenge revealed that the birds exhibited decreased oxygen consumption, carbon dioxide expiration, and heat production, which contrasts with the elevated basal metabolism observed under acute heat stress conditions of experiment 1. To corroborate these findings, it was earlier demonstrated by Patton et al., 2018) that heat exposure elevated basal respiration and respiration, whereas LPS treatment reduced peak respiration and generated energy deficits of skeletal muscle myotubes. These differential outcomes were attributed to different functional metabolic pathways affording cross-tolerance, since heat exposure increases energetic stress, mitochondrial biogenesis, and metabolic capacity, whereas LPS challenge reduced reliance on basal glycolytic function to better maintain cellular metabolic capacity. Interestingly, challenging broilers with acute LPS treatment decreased the RQ, suggesting an increase in fatty acid oxidation or gluconeogenesis to meet energy requirements regardless of dietary effect (McKee et al., 1997). This work demonstrates the capacity of heat stress and LPS challenge to dysregulate whole body metabolism, which will help to inform future research on stress induced responses and their regulation of whole-body metabolic capacity.

As important metabolic substrates, amino acids and their metabolites play significant functions in physiological processes, including protein synthesis, mitochondrial biogenesis, energy metabolism, and thermotolerance (Chowdhury et al., 2021; Ling et al., 2023 As a functional amino acid, L-citrulline is known for its role in arginine synthesis, anti-inflammation, anti-oxidation, gut modulation, and thermoregulatory effects in poultry (Ma et al., 2025; Uyanga et al., 2022a). In this study, providing broiler chickens with dietary L-citrulline reduced the core body temperature by ~0.43°C compared to the unsupplemented birds under acute heat stress, although without statistical significance. L-citrulline supplementation effectively reversed LPS-induced febrile response by reducing the core body temperature (~0.48°C) during acute LPS challenge. Under chronic conditions, similar hypothermic effects were evident with the L-citrulline diet decreasing the surface temperature of broilers and further interacting with time to initiate a temporal decline in rectal temperatures of L-citrulline-fed birds relative to the unsupplemented birds. However, dietary L-citrulline could not reverse the impacts of chronic LPS challenge on the rectal, surface, or core body temperatures. Prior studies have proven that L-citrulline supplementation is effective in assisting poultry cope with the negative impacts of heat stress by initiating reductions in body temperature and affording thermotolerance to birds (Uyanga et al., 2021, 2022d). This study provides further evidence of L-citrulline-induced hypothermia, although it expressed varying effectiveness in attenuating stress induced hyperthermia under acute and chronic conditions.

L-citrulline has been reported as an effective supplement that would preserve mitochondrial integrity and mitigate heat-induced oxidative stress and mitochondrial dysfunction (Yu et al., 2023). In the present study, findings from both experiments 1 and 2 revealed that dietary L-citrulline supplementation acted independently of stress condition to increase the oxygen consumption, carbon dioxide expiration, and heat production of broiler chickens relative to the unsupplemented birds. The increased respiratory gases and heat production observed in L-citrulline-fed broilers may have resulted from an increased metabolic activity, since it has been established that citrulline stimulates mitochondrial biogenesis and muscle protein synthesis (Allerton et al., 2018; Jourdan et al., 2015). This aligns with earlier reports that L-citrulline is an important regulator of cellular energy metabolism, with the capacity to regulate mitochondrial biogenic genes and the activities of the respiratory chain complex (Uyanga et al., 2022d). Han et al., 2018) also demonstrated a similar sequence of events for L-leucine, a well-known proteinogenic amino acid, since the *in ovo* administration of L-leucine stimulated embryonic heat production and metabolic rate, which could be related to its role in increasing mitochondrial activity and protein synthesis.

Contrarily, when L-citrulline was co-administered with a stressor (LPS/heat-stress), it lowered the respiratory quotients of both the LPS-challenged and heat-stressed birds less than during normal conditions. Heat stress and LPS challenge both contribute to immunosuppressive effects, which is an energy-consuming process that may alter energy supply and demand in poultry (Yang et al., 2024). L-citrulline-fed broilers under heat stress and LPS challenge had RQ values of 0.73 and 0.66, respectively. Generally, the RQ values reflect the primary substrate being metabolized and the metabolic shifts from carbohydrate metabolism to protein and lipid catabolism or otherwise. Therefore, the lower RQ values of ~0.70 in L-citrulline birds under heat stress and LPS challenge suggest a higher metabolic rate since the body fat and protein were mobilized as metabolic fuel to generate energy, probably due to mediate stress induced dysregulation and immune disturbance (Jiang et al., 2025).

L-citrulline has been well documented as an effective precursor for arginine and NO generation through the citrulline - NO cycle (Bailey et al., 2015). Previous studies demonstrated that dietary supply of L-citrulline at 0.25 to 1.00% would elevate the circulating NOS activity, NO levels, arginine, and citrulline concentrations in chickens (Uyanga et al., 2020). In the present study, L-citrulline did not induce any significant change in NO levels during acute conditions. However, its prolonged administration significantly reduced the circulating NO levels under chronic conditions. This corresponded with the reduction in iNOS activity, which was consistently observed under both acute and chronic L-citrulline feeding and chronic LPS administration. Studies have shown the temporal changes with stress induced iNOS expression, which may be stimulated as a necessary pro-inflammatory response during exposure to challenge conditions, or inhibited as a protective mechanism to attenuate its overexpression under pathophysiological conditions (Chen et al., 2006; Clarke et al., 2019). As such, the iNOS inhibition may be a necessary protective mechanism to limit inflammatory mediator production and the decline of its associated signaling cascade.

Transient receptor potentials (TRP) are a family of membrane proteins that act as ion channels, involved in detecting and responding to various environmental and internal stimuli such as thermal, nociceptive, mechanical, chemical, and light sensation (Kashio, 2021). Among the temperature-sensitive TRP channels are the heat-activated channels (TRPV1-4) and cold-activated channels (TRPM8 and TRPA1), which are expressed in various tissues, including the central nervous system, skin, and other organs (Hsu and Yoshioka, 2015). These thermo-TRPs are involved with temperature sensitivity and intervene in the maintenance of homeostasis and thermoregulation in broiler chickens (Wang et al., 2023). To gain insights into the functionality of these thermoreceptors and their role in maintaining the body temperature of animals, or susceptibility to thermal stress, the mRNA expression of both cold-sensing and heat-sensing channels was investigated in major thermosensing organs of poultry, the skin and hypothalamus.

Contrary to expected outcomes, the acute heat stress challenge did not influence the skin and hypothalamic expression of TRP ion channels, except for hypothalamic TRPV1 expression, which was upregulated under acute heat stress conditions. This finding aligns with established reports that heat stress initiates hyperthermia, which leads to a significant upregulation of TRPV1 expression in the brain (Aghazadeh et al., 2021). TRP ion channels in skin are crucial for thermosensation and the maintenance of thermal balance and homeostasis (Hsu and Yoshioka, 2015). Under acute conditions of experiment 1, the dietary effect of L-citrulline was evident as its supplementation upregulated the skin TRPV1 and TRPV3 expression, whereas it tended to downregulate hypothalamic TRPV3 and TRPM8 expressions. L-citrulline’s influence on TRP ion channels may be related to its renowned role in activating NO synthesis and bioavailability (Chen et al., 2023). Evidence suggests that TRPV ion channels contribute to vasodilation through NO-dependent pathways, regulating the normal and pathological functions (Baylie and Brayden, 2011). These patterns of L-citrulline induced changes suggest tissue-specific TRP responses, which necessitate further investigation as studies on TRP ion channels in poultry are gradually emerging.

Under chronic LPS challenge, both the skin TRPV3 and TRPA1 expressions were downregulated in the control-fed broilers, and the provision of an L-citrulline diet also decreased the skin TRPA1 expression. Previous reports have identified that the expression of TRPA1 channel ions extends far beyond the sensory nerves in the skin, for them to act as nociceptive sensors and potentiate the inflammatory process in non-neuronal cells (Gouin et al., 2017). Plausibly, the deactivation of TRPA1 expression by L-citrulline under LPS challenge may occur as an anti-inflammatory response; however, further studies may be necessary to ascertain this.

## 5 Conclusion

In conclusion, this work demonstrates that L-citrulline may be a viable anti-stress agent in poultry as it can initiate thermoregulatory and metabolic responses necessary to re-establish homeostasis and ensure survival. While L-citrulline may be provided under stress conditions, it can also act independently to influence body temperature regulation, thermosensation, and basal metabolism. The findings from this work provide fundamental information that will bridge the knowledge gap on L-citrulline’s potential as a thermoregulatory agent, evoking questions for future investigations into the physiological role of thermosensitive ion channels in poultry species.

## Data Availability

The datasets presented in this study can be found in online repositories. The names of the repository/repositories and accession number(s) can be found in the article/**Supplementary Material**.
