# Supplementary material for: Respiratory efficiency and thermoregulatory responses of L-citrulline-supplemented broiler chickens under acute and chronic stress conditions
Source: Front Physiol. 2026 Apr 21;17:1785584. doi: 10.3389/fphys.2026.1785584 (PMC13139027; doi:10.3389/fphys.2026.1785584)
Supplement: Supplementary file 1 [file SupplementaryFile1.docx]

**Supplementary File**


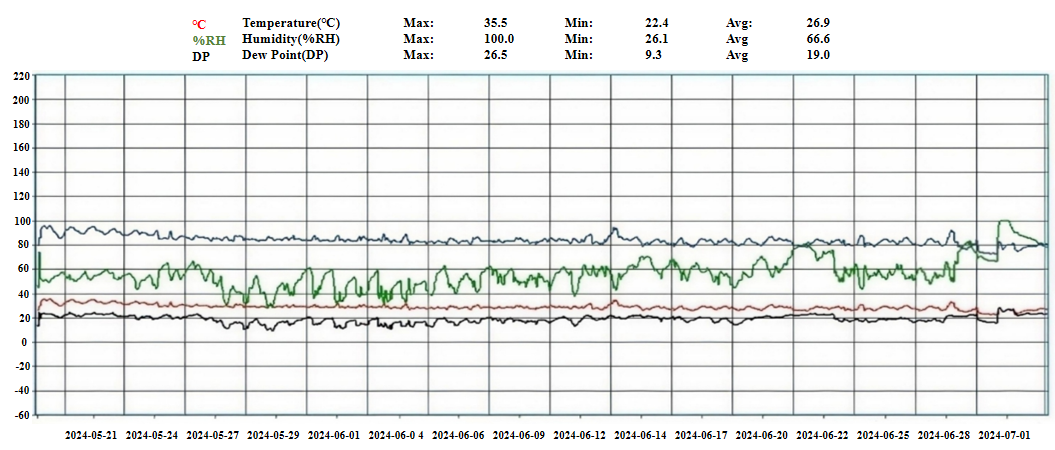


Supplementary Figure 1: Summary of environmental conditions during the study period
